# *Entamoeba histolytica*‐associated proctitis and ileitis mimicking Crohn's disease—A case report

**DOI:** 10.1002/ccr3.6833

**Published:** 2023-05-20

**Authors:** Thomas Griemert, Ekkehard Siegel, Moritz Brandstetter, Beate K. Straub, Andreas Kreft, Peter R. Galle, Martin F. Sprinzl

**Affiliations:** ^1^ Department of Internal Medicine I University Medical Center of the Johannes Gutenberg University Mainz Germany; ^2^ Institute of Microbiology University Medical Center of the Johannes Gutenberg University Mainz Germany; ^3^ Institute of Pathology University Medical Center of the Johannes Gutenberg University Mainz Germany

**Keywords:** amebic enteritis, MSM, proctitis, sexually transmitted disease

## Abstract

We report about a proctitis and ileitis terminalis, leading to the misdiagnosis of Chron's disease, in a male patient who has sex with men. Molecular multiplex analysis identified *Entamoeba histolytica* as the underlying cause. We provide diagnostic images, clues and pitfalls for diagnosis of *E. histolytica* associated proctitis.

## Abstract 2

Diagnosis and treatment of sexually transmitted proctitis should cover C. trachomatis, N. gonorrhoeae, and Treponema pallidum. Treatment failure and recurrent intestinal symptoms require detailed clinical history and screening for *E. histolytica*.

## 1 BACKGROUND

Sexually transmitted proctitis is a common finding among men who have sex with men (MSM). Sexually transmitted proctitis typically results from *Chlamydia trachomatis*, *Neisseria gonorrhoeae*, and *Treponema pallidum* infections, which guides initial diagnostic work up and antibiotic therapy.^1^

Here we report about a treatment refractory proctitis, which led to the misdiagnosis of a chronic inflammatory bowel disease. Molecular multiplex analysis eventually identified *Entamoeba histolytica* as the underlying cause. The following case report will provide a detailed clinical work‐up and discusses clues, which could guide differential diagnosis of *E. histolytica*‐associated proctitis.

## 2 INITIAL CASE PRESENTATION

We report about a 53‐year‐old male patient, who attended the infectious disease clinic for further assessment of an ulcerative proctitis. A *Brachypsira aalborgii* positive stool culture had previously prompted a course of metronidazole for 7 days, which achieved a transient clinical improvement for three weeks.

At first presentation in August 2018, the patient reported about rectal tenesmus, leading to frequent small volume bowl movements, hematochezia, and mucous discharge. Dysurea, pollakiuria, and urethral discharge were denied. He reported about recent homosexual contacts with a partner of Chinese descent. External ano‐genital examination did not reveal pathological findings.

Infectious disease screening was negative for HIV, viral hepatitis, and *Treponema pallidum*. Urethral and anal swaps did not identify *N. gonorrhoeae* and *Trichomonas vaginalis*. Serology for *Chlamydia trachomatis* (C. trachomatis: IgG >200.0 AU/ml, IgA =58.8 AU/ml) was positive.

**FIGURE 1:**
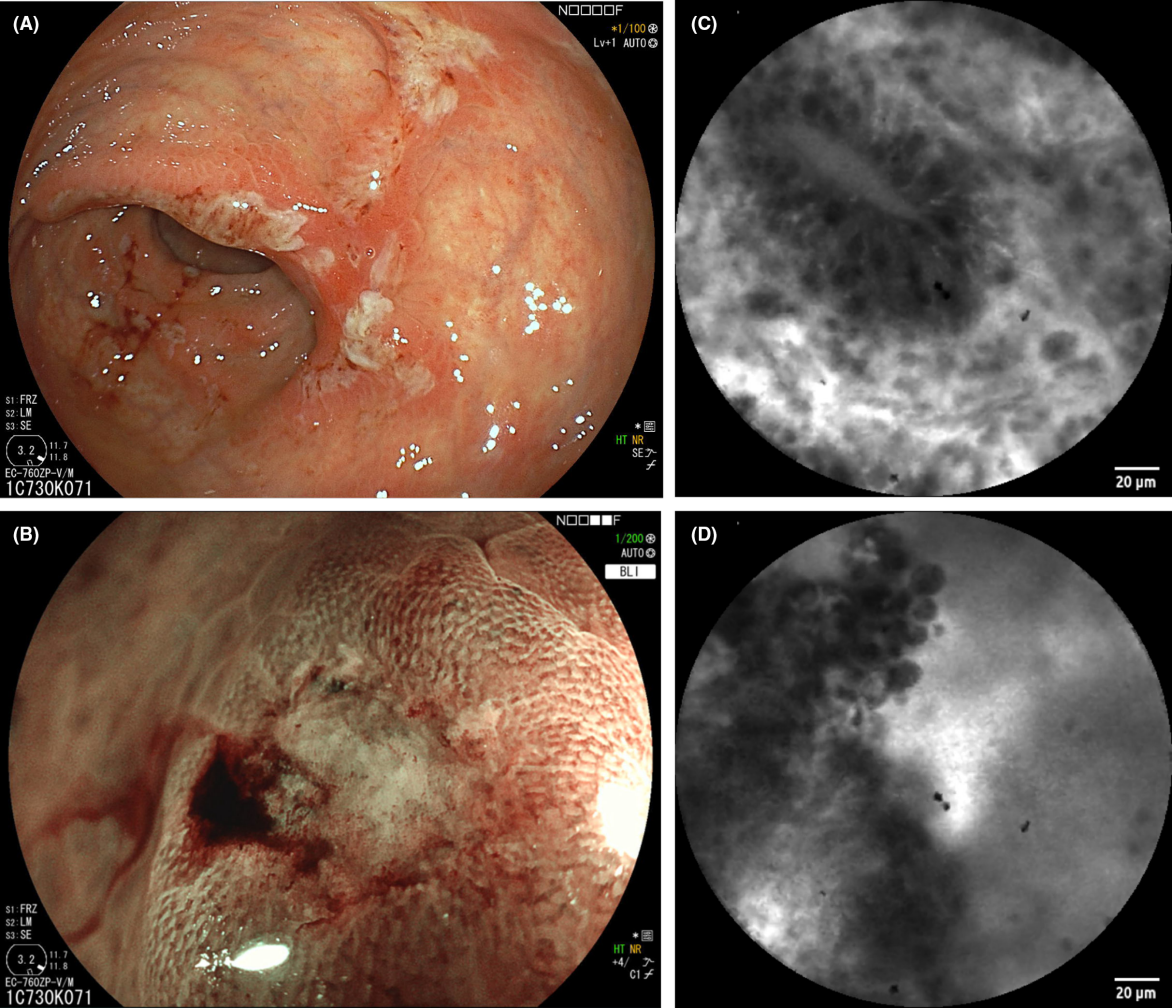
Endoscopy and confocal endomicroscopy. Sigmoidoscopy showing exsudative mucosal ulcerations (A) of the rectum. Spontaneously bleeding ulcerations (B) as observed by high‐resolution endoscopy (100‐fold magnification, blue laser imaging endoscopy, Fuji, Japan). Confocal microscopy after flourescein application shows elongated crypts and effusions (C) as well as mucosal destructions at the ulcer margine (D) (CellVizio, Paris, France).

Proctoscopy confirmed an ulcerative proctitis with vulnerable mucosa (Figure 1A/B) and confocal endomicroscopy showed mucosal edema, capillary leakage, and focal enterocyte destruction (Figure 1C–E). Histopathology from this examination also showed mild active inflammation with polar crypts and increased eosinophilic infiltrates. No signs of malignancy were found. Polymerase chain reaction (PCR) from the colorectal biopsy was positive for *Chlamydia trachomatis*, whereas PCR for *N. gonorrhoeae*, cytomegalovirus, and herpes simplex I/II remained negative.

Diagnosis of Chlamydia proctitis prompted a 7‐day course of Doxycyclin (100 mg bid), which led to clinical improvement but failed complete resolution of symptoms. After recurrent episodes of rectal tenesmus, and frequent bloody bowl movements an additional 21‐day course of Doxycyclin (100 mg bid) as well as a single dose ceftriaxon (2 g IV qd) administration followed without clinical improvement. Additional courses of metronidazole for two weeks provided transient symptom relieve. Our patient remained sexually abstinent throughout this time‐period, and follow‐up examinations did not identify any sexually transmitted infections (e.g., *Chlamydia trachomatis*, *N. gonorrhoeae* and *Treponema pallidum*).

## 3 CLINICAL FOLLOW‐UP AND FINAL DIAGNOSIS

**FIGURE 2:**
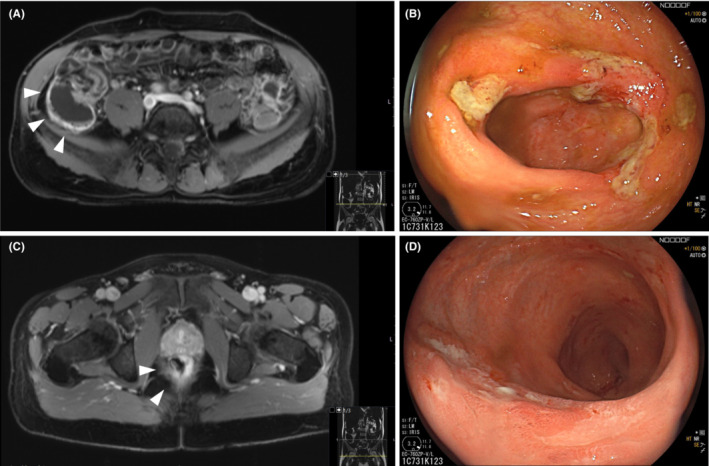
Hydro‐MRT and pancolonoscopy. Hydro magnet resonance tomography showing contrast enhancement of the coecal pole (A) and rectum (B) indicating inflammation in these areas (white arrows) as well as a prominent ileo‐coecal valve. Corresponding endoscopy shows ulcerations and inflammation of the rectum (C) and caecum (D).

After the ulcerative proctitis remained refractory to antibiotic therapy Mesalazin and Budesonide enemas were prescribed and our patient was referred to the chronic inflammatory bowel disease (IBD) clinic for further assessment in June 2020. Colonoscopy revealed mucosal ulcerations affecting the rectum, caecum, ileocaecal valve and terminal ileum. Histopathologic findings were unspecific, but principally in line with an IBD. Crohn's disease was suspected due to the typical discontinuous inflammatory distribution pattern (Figure 2A/B). Hydro magnet resonance tomography confirmed contrast enhancement in the rectum and caecal region. The examination did not identify fistula, stenosis or additional small intestinal involvement. However, increased contrast uptake of the prostate indicated an ongoing inflammation in this area (Figure 2C/D). Following a negative screening for *Mycobacterium tuberculosis*, hepatitis B and *Clostridium difficile*, he received a systemic anti‐inflammatory treatment with glucocorticoids since June 2020. However, fecal Calprotectin concentrations remained elevated (322–438 μg/g, norm <50 μ/g) under treatment and symptoms did not resolve. Treatment escalation with anti‐TNF‐α antibodies (Adalimumab, continuous dose 40 mg bi‐weekly) started after one month, due to the glucocorticoid‐dependent clinical course. Clinical treatment failure after six months of anti‐TNF‐α blockage prompted a second switch to anti‐IL‐12/23 antibodies (Ustekinumab, 90 mg every 8 weeks). Despite treatment escalation, clinical activity and fecal calprotectin (319 μg/g, norm <50 μ/g) remained unresponsive for 11 months. During this period, conventional bacterial stool cultures, mycobacterial cultures, C. difficile testing, and molecular cytomegalovirus diagnostics remained negative.

**FIGURE 3:**
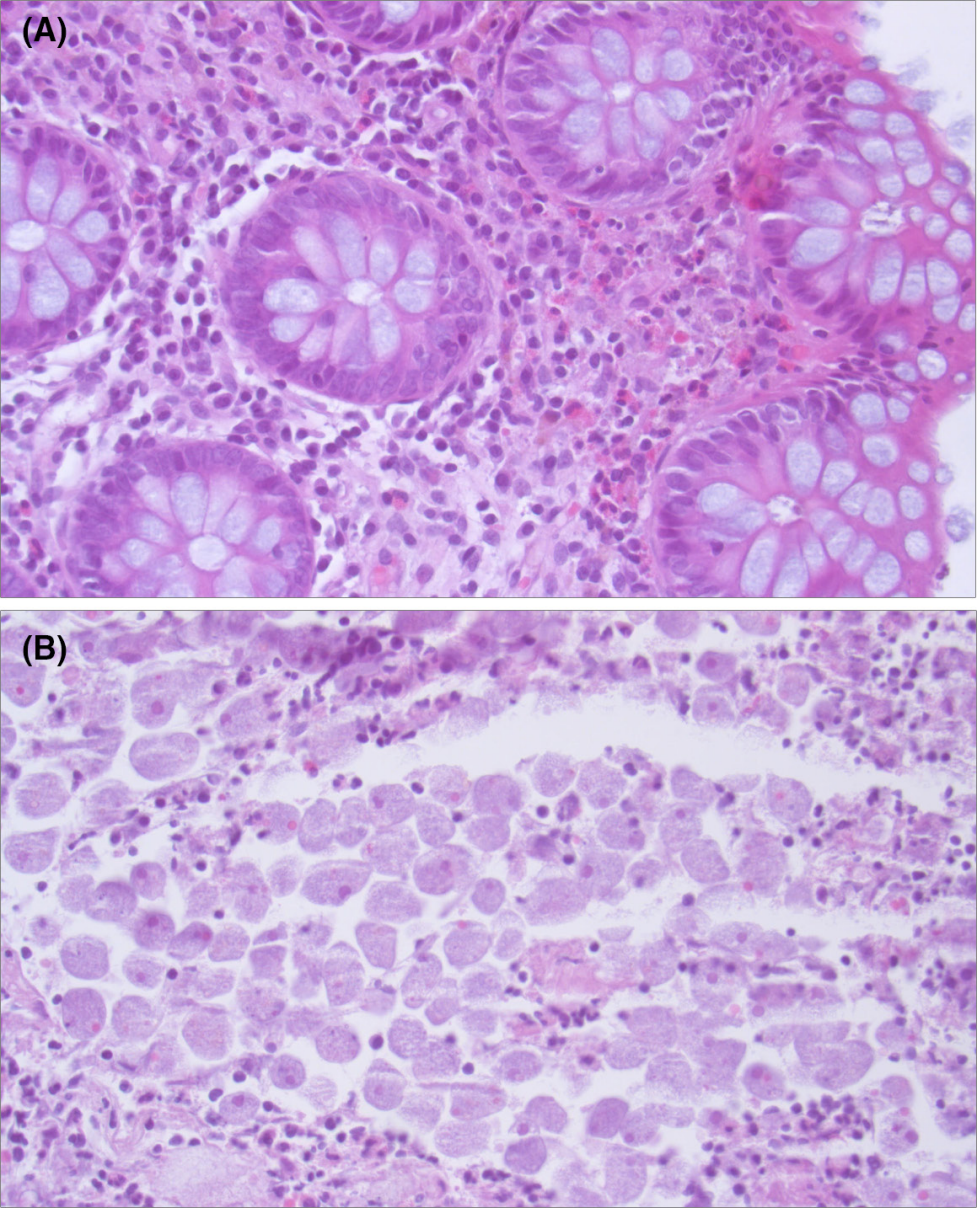
Histopathology. Hematoxylin–eosin staining of colon biopsies (400‐fold magnification). (A) Chronic and acute colitis with prominent eosinophilic infiltrates. (B) *Entamoeba histolytica* trophozoites in the stool.

We finally performed a multiplex stool analysis to identify occult infectious causes of chronic colitis (FilmArray, gastrointestinal panel, BioFire Diagnostics, LLC).^2^ The multiplex‐PCR screening of 22 different enteric pathogens (bacteria, viruses, and protozoae) identified *Entamoeba histolytica*. Histopathology at this time point identified a colitis and mucosal ulcerations with eosinophilic infiltrates, yet no parasites adherent to the colon mucosa (Figure 3A). Microscopy of stool demonstrated multiple *E. histolytica* cysts with inoculation of erythrocytes in confirmation of the diagnosis of amebic colitis (Figure 3B). A course of metronidazole (500 mg tid) for 10 days followed by Paromomycin (500 mg tid) for 10 days led to symptom resolution. Half year later, our patient was still asymptomatic.

## 4 DISCUSSION

The outlined case highlights *Entamoeba histolytica* as cause of sexually transmitted proctitis, which shared clinical features of IBD. Despite a directional sexual history, the underlying cause of proctitis was not recognized, especially since a chlamydial infection was identified as a typical cause of proctitis in the first place. The misleading discontinuous rectal and ileocaecal inflammation further suggested Crohn's disease. The differential diagnosis of IBD from amebic colitis is a recognized challenge in tropical regions but remains uncommon in temperate climates. Therefore, standard diagnostic procedures do not specifically address *Entamoeba histolytica* during routine work‐up.

Several lines of evidence, however, could have led to the diagnosis of *E. histolytica*. The mode of transmission could have guided to the diagnosis of *E. histolytica*, as it has been identified in up to 20% among men who have sex with men (MSM).^3^ In a German case series, *E. histolytica* was found in stool samples of 16.3% mostly asymptomatic MSM patients.^4^

An anamnestic link to an endemic region of *E. histolytica* would indicate amebic enteritis. In our case, the patient himself did not report any travel history. However, his sexual partner originated from China, where an average *E. histolytica* seroprevalence of 6.2% exists in the general population.^5^ *E. histolytica* seroprevalence even reaches 41% in Chinese risk populations practicing receptive anal sex (odds ratio: 2.03; 95% CI: 1.22 3.37).^6^ The fact that the sexual partner of our patient was reportedly asymptomatic is conclusive, as *E. histolytica* infection is self‐resolving in 90% and asymptomatic carriers may transmit the infection.^3^, ^4^, ^7^

Clinical response to metronidazole, which is effective against *E. histolytica*, provided additional evidence for amebic enteritis. Even recurrence of amebic enteritis after metronidazole monotherapy is seen, as persistent amebic cysts may lead to treatment failure.^8^

Initial examination, however, identified *Brachypsira aalborgii*, a known human pathogen of intestinal spirochetosis. Symptomatic infection typically occurs in MSM risk populations.^9^, ^10^ As intestinal spirochetosis responds well to metronidazole,^10^, ^11^ we believed that a coinfection with a resistant pathogen caused symptom recurrence, particularly after *C. trachomatis* was confirmed in rectal biopsies. Metronidazol, on the other hand, can also transiently improve Crohn's disease activity, which further hampered correct interpretation of treatment response.^12^, ^13^

Clinical presentation of IBD and amebiasis are quite similar, which carries the risk of misdiagnosis. The endoscopic picture of small irregular ulcers surrounded by normal mucosa is a typical feature of amebiasis and may also be found in Crohn's disease.^14^ Amebic enteritis frequently affects the caecum and rectum in 56% at the same time, which is an additional misleading feature consistent with Crohn's disease.^15^ Pathological diagnosis of amebic enteritis is also difficult because biopsy material may not represent pathognomonic features. Focal eosinophilic infiltrates are not discriminative as they may be present in both IBD and amebic enteritis.^16^, ^17^ In our case, small forceps biopsies missed typical flask‐shaped ulcers of amebic enteritis. Colon biopsies did also not show *E. histolytica* trophozoites in a routine periodic acid–Schiff staining during initial histopathological assessment. *E. histolytica* trophozoites typically locate within necrotic tissue on the mucosa surface, which could detach from the biopsy probe, making them difficult to detect.^15^ Indeed, retrospective assessment identified numerous *E. histolytica* trophozoites in the stool, but not in the colon biopsies themselves (Figure 3B).

The outlined case highlights pitfalls of *E. histolytica* diagnosis, which could be overcome by a standardized approach to sexually transmitted proctitis. *C. trachomatis*, *N. gonorrhoeae*, and *Treponema pallidum* should be covered by initial diagnostic work‐up and empiric treatment, as these entities are the most prominent causes of proctitis or rectal lesions in risk populations, respectively.^1^, ^18^, ^19^ Following treatment failure and inconclusive diagnostic findings, we strongly recommend to screen for other pathogens including *E. histolytica*. PCR‐based tests have proven a very high specificity and sensitivity superior to conventional diagnostic test, which could guide targeted therapy.^2^

## AUTHOR CONTRIBUTIONS

**Thomas Griemert:** Investigation. **Ekkehard Siegel:** Investigation. **Moritz Brandstetter:** Investigation. **Beate K. Straub:** Investigation; writing – review and editing. **Andreas Kreft:** Investigation. **Peter R. Galle:** Resources. **Martin F. Sprinzl:** Conceptualization; investigation; writing – original draft.

## FUNDING INFORMATION

Not applicable.

## CONFLICT OF INTEREST

All authors declare that they have no conflicts of interest in the context of this work.

## ETHICAL APPROVAL

Not applicable.

## CONSENT

Written informed consent was obtained from the patient to publish this report in accordance with the journal's patient consent policy.

## CONSENT TO PARTICIPATE

Informed consent was obtained from the patient included.

## Data Availability

Data sharing not applicable to this article as no datasets were generated or analysed during the current study.
